# Fast Hydrogenation and Dehydrogenation of Pt/Pd Bimetal Decorated over Nano-Structured Ag Islands Grown on Alumina Substrates

**DOI:** 10.3390/s19010086

**Published:** 2018-12-27

**Authors:** Md Habibur Rahaman, Usman Yaqoob, Hyeon Cheol Kim

**Affiliations:** School of Electrical Engineering, University of Ulsan, 93 Daehak-ro, Nam-gu, Ulsan 44610, Korea; habibiiuceee@gmail.com (M.H.R.); usmanyqb3@gmail.com (U.Y.)

**Keywords:** hydrogenation, dehydrogenation, Ag nano islands, Pt/Pd bimetal

## Abstract

This study reports the fast hydrogenation and dehydrogenation of ultra-thin discrete platinum/palladium (Pt/Pd) bimetal over nano-structured Ag islands grown on rough alumina substrate by a RF magnetron sputtering technique. The morphology of Ag nanoislands was optimized by RF magnetron sputtering and rapid thermal annealing process. Later, Pt/Pd bimetal (10/10) nm were deposited by RF magnetron sputtering on the nanostructured Ag islands. After the surface morphological optimization of Ag nanoislands, the resultant structure Pt/Pd@Ag nanoislands at alumina substrate showed a fast and enhanced hydrogenation and dehydrogenation (20/25 s), response magnitude of 2.3% (10,000 ppm), and a broad detection range of 500 to 40,000 ppm at the operating temperature of 120 °C. The superior hydrogenation and dehydrogenation features can be attributed to the hydrogen induced changes in the work function of Pt/Pd bimetal which enhances the coulomb scattering of percolated Pt/Pd@Ag nanoislands. More importantly, the atomic arrangements and synergetic effects of complex metal alloy interfacial structure on Ag nanoislands, supported by rough alumina substrate incorporate the vital role in accelerating the H_2_ absorption and desorption properties.

## 1. Introduction

For decades, as being one of the most extensively used gases, hydrogen has been of interest to create a great economy in the fields of clean energy transportation and system, petroleum product refining, fuel source, power generation, and energy storage [1,2,3,4]. However, the use of hydrogen is associated with various safety-related issues as it’s a colorless and odorless gas with explosive properties at concentrations over 4% [5]. Thus, it needs an accurate measuring system, by which hydrogenation phenomena can be accurately detected within very short times under different conditions [5]. Recently, lots of studies and experiments were performed by researchers studying the smooth hydrogenation and dehydrogenation of various catalytic metal oxides system such as In_2_O_3_, SnO_2_, ZnO, TiO_2_, and WO_3_ [6,7,8,9]. Recently, Xue et al. showed a mesoporous SnO_2_ modified layers- based hydrogen gas sensor which requires a 300 °C working temperature for a response and recovery time of 82/451 s [6]. However, their high temperature requirements increase the power consumption which are not desirable for efficient hydrogenation and dehydrogenation systems [5,6,7,8]. Furthermore, the slow response and recovery for the aforementioned metal oxides may affect the safety issue. To address these issues, researchers have deployed palladium (Pd) as a metal catalyst which has a high selectivity with a good hydrogen gas absorption rate at lower temperature [10,11]. Lewis et al. reported the fundamental mechanism of the hydrogen absorption and desorption process in Pd-based thin films where a linear increase in the electrical resistance of the Pd-H system as a function of H content was shown [12]. Choi et al. reported 0.5 wt % Pd/SnO_2_ which shows a response time of 40 s to 1% hydrogen at 550 °C that still represents a high working temperature [13]. Liewhiran et al. observed high sensitivity towards hydrogen absorption in a Pd/ZnO-based sensor though it needed 400 °C with a slower (min) recovery time [14]. Kiefer et al. showed nanogap-based Pd films on polyamide and found the limit of detection was 2% on room temperature with a response and recovery time of 52/122 s [15]. However, during the absorption and desorption process, the mechanical embrittlement and the hysteresis effect at higher hydrogen concentration make sensing ability of the Pd thin film-based hydrogen sensor unstable [16,17,18,19,20,21]. Several kinds of Pd nanostructures such as nanowires, nanochains, nanoflowers, nanotubes, and nanocomposites show improved results in terms of stability and power consumptions [22,23,24,25,26,27]. Keifer et al. also showed a Pd microwire-based hydrogen sensor with a response and recovery time of 362/303 s at 4% hydrogen gas with a minimum detectable range of 2% [28]. Yang. et al. observed a 2 ppm lower limit of detection of hydrogen by synthesizing 33 nm height, 47 nm width Pd nanowire, 25 nm height, 85 nm width Pd nanowire, although the response and recovery time was 30/100 s (4% H_2_) and 100/200 s (4% H_2_) [29,30]. Similarly like Pd, platinum (Pt) nanoparticles have good hydrogen adsorption and desorption properties, although Pd nanoparticles have higher reactivity and durability [31]. Thus, the combination of Pt and Pd nanoparticles could achieve synergistic effects to enhance the performance of the H_2_ sensor [28,31]. Moreover, Pt-Pd is known to show better hydrogenation properties in comparison to Pd [28]. Peng et al. showed that combining Pd with Pt at nanoscale label on reduced graphene oxide enhances the hydrogenation and dehydrogenation properties where 3% hydrogen was detected at room temperature although a slower response (~5 min) and recovery time (>20 min) was observed [31]. However, these structures require a complex fabrication process along with conductive graphene derivatives which might face some agglomeration effects. Thus, it is highly desirable to design a simple cost effective structure with highly catalytic Pt/Pd metal alloy for enhanced hydrogenation and dehydrogenation processes. Ag is a cost effective and light weight metal which can provide a better catalytic interfacial alloy formation with Pd that has more solubility and permeability towards hydrogen gas [32,33]. However, only Pd-Ag alloy couldn’t satisfy the fast hydrogenation and dehydrogenation process demands [32,33]. So more comprehensive studies are needed to optimize the catalytic properties.

In this present study, for the very first time, we report the hydrogenation and dehydrogenation of nano-sized Pt/Pd bimetal decorated over Ag nanoislands grown on an alumina substrate. Thin Ag films were deposited by ultra-high vacuum RF magnetron sputtering followed by a rapid thermal annealing process for inducing the formation of nanoisland morphological structures. Pt/Pd bimetal was also deposited by RF magnetron sputtering on the Ag nanoislands to form a capping layer. The nanostructured Ag islands morphology provides a higher interfacial coverage with the catalytic bimetal of Pt/Pd. Furthermore, it is expected that, the island structure would provide a percolated conductive pathway by increasing the connectivity with the bimetal at the nanoscale level. The as-fabricated structure was used as to observe the resistance change for detection of hydrogenation and dehydrogenation processes.

## 2. Experimental Section

Fast hydrogenation and dehydrogenation, higher response magnitude, broader range of detection, good repeatability, and less power consumption are important factors for hydrogen sensing, storage, fuel cell-based electronics. To meet these requirements, we developed and fabricated Pt/Pd bimetal decorated over percolated nanostructured Ag nanoislands on a rough alumina substrate by RF magnetron sputtering. To optimize the Ag nanoisland structure, we grew different nanosized Ag particles by varying the deposition conditions and applied a rapid thermal annealing process to connect the particles by a coalescence mechanism. This modified nano- structured particles after coalescence showed islands structure which later helps to provide a percolated islands structure with enhanced Ohmic conduction. This percolating channel was greatly supported by the Pt/Pd bimetal to be deposited for hydrogen permeation and release. Pt/Pd bimetal with high surface to volume ratio was deposited in a discrete film manner over the Ag nanoislands. Finally, we explored the hydrogenation and dehydrogenation properties of this new material by applying a bias voltage in a realistic gas environment, in which fast response and recovery, and changes in hydrogen concentrations were found.

### 2.1. Device Fabrication

**Figure 1:**
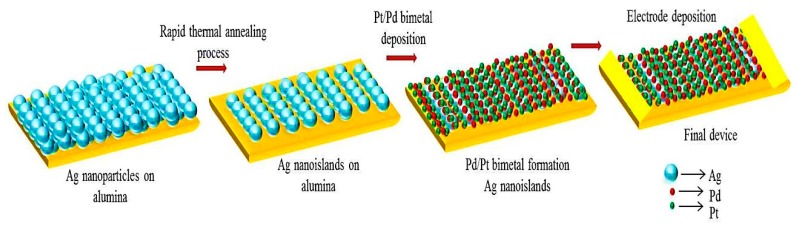
Device fabrication.

A highly rough electrically insulated alumina substrate (10 × 5 mm^2^) was cleaned in acetone and isopropanol for 5 min followed by ultrasonication for 5 min. To make the Ag nanostructured islands over alumina, RF magnetron sputtering with an ultra-high vacuum chamber (RF power 130 Watt) was utilized for depositing nanosized Ag particles. To form a percolation supportive morphology and develop a nanoisland structure, a rapid thermal annealing process was sub-sequentially followed. Different temperatures such as 200, 300, 350, 400, 500, and 600 °C along with an argon gas environment (vacuum condition) were applied in the annealing process. Optimized nanostructured Pt/Pd bimetal with high surface to volume ratio was sequentially deposited in a discrete thin film manner on the Ag nanoislands by the RF magnetron sputtering system. The percolated Ohmic conduction of the as-prepared bimetal/nanoislands/alumina substrate was analyzed in the probe station system by coating with two thick silver electrodes parallel to each other with 2 mm distance. The whole fabrication process is shown in Figure 1.

### 2.2. Characterization

The surface morphologies of Ag nanoparticles on the alumina substrate with different annealing conditions were analyzed using field emission scanning electron microscopy (FESEM; JSM JEM–7600F, JEOL, Ulsan, Korea). Nanostructured Ag nanoislands roughness and RMS grain size was checked using 3D atomic force microscopy (AFM). Water droplet contact angles were measured by contact angle goniometry (DSA 100 drop shape analyzer, Kruss, Ulsan, Korea) using the sessile drop method at room temperature. Deionized water droplets (about 3 μL) were dropped on the thin film surfaces using a micro-syringe. Pt/Pd bimetal and their nanostructured discrete thin film distribution were studied with a JEOL JEM-2010F energy dispersive spectrometer (EDS). The structural properties were investigated using an X-ray diffractometer (XRD, Ultima IV, Rigaku, Ulsan, Korea) with Cu Kα (λ = 0.154 nm) radiation over a 2θ scanning range of 10–90°. The chemical compositions were checked by X-ray photoelectron spectroscopy (XPS) using Al Kα radiation as the X-ray source. Photo-luminescence (PL) spectra were obtained on a LS 55 setup (Perkin Elmer, Ulsan, Korea). A SCS-4200 probe station (Keithly, Ulsan, Korea) was used for measuring the resistance changes of the as-prepared devices.

### 2.3. Sensing Measurement

The as-prepared device was mounted inside of a chamber, and the Keithly probe station with a bias voltage fixed at 1 V was used to check the ohmic conduction and resistance. A computerized mass flow controller system (GMC 1200, ATOVAC, Ulsan, Korea) was used to change the concentration of H_2_ gas in synthetic air (Deokyang Co., Ltd., Ulsan, Korea). Gas mixtures with different H_2_ concentrations were delivered to the chamber at a constant flow rate of 200 sccm. The gas chamber was supplied with synthetic air between each H_2_ pulse to allow the device surface to return to atmospheric conditions. The gas concentration was controlled and measured using the following equation:

$$\mathrm{Desired}\;{\mathrm{gas}\;}_{\mathrm{con}.}\;(\mathrm{ppm})=\frac{{\mathrm{Flowrate}}_{\mathrm{gas}}}{{\mathrm{Flowrate}}_{\mathrm{gas}}+{\mathrm{Flowrate}}_{\mathrm{synthetic}\;\mathrm{air}}}\times \mathrm{Supplied}\;{\mathrm{gas}\;}_{\mathrm{con}.}\;(\mathrm{ppm})$$

The sensor response magnitude was defined as:

$$S\;(\%)=\frac{R_{g}-R_{a}}{R_{a}}\times 100$$

where *R_g_* and *R_a_* is the resistances of the sensor in hydrogen gas exposure and air exposure at certain concentrations, respectively. The response and recovery time of the as-prepared device was defined as the time taken to reach 90% of the total resistance change and the time needed for the resistance to reach its initial value.

## 3. Results and Discussion

### 3.1. Materials Structure and Morphology

**Figure 2:**
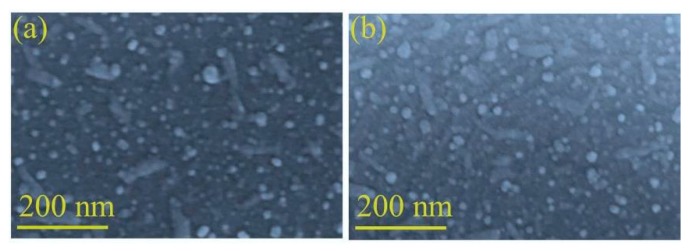
Surface morphology of Ag nanoparticles without annealing (**a**) 18.50 nm (**b**) 25 nm.

In order to grow Ag nanoislands, two sizes of (18, 25 nm) Ag nanoparticles were deposited on alumina substrate by varying the condition in RF magnetron sputtering. Figure 2a,b shows the surface morphology of non-annealed Ag nanoparticles. Highly dense and agglomerated nano-particles can be observed on the surface which represents an irregular formation of nanoparticles.

**Figure 3:**
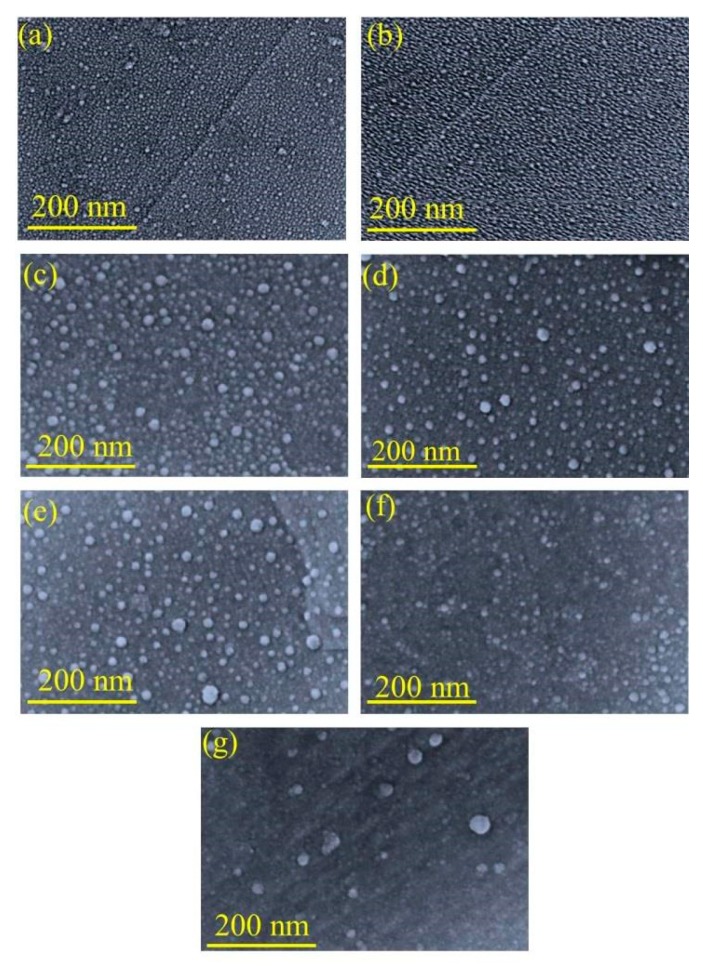
Surface morphology of Ag nanoparticles at (**a**) 200 °C; (**b**) 300 °C; (**c**) 350 °C; (**d**) 400 °C; (**e**) 500 °C; (**f**) 550 °C; (**g**) 600 °C.

A thicker NPs deposition might not be enough to form a continuous film as atoms deposited from the vapor phase undergo a series of kinetic processes, including thermal accommodation onto the substrate, surface diffusion of the atoms on the surface, dimer formation to initiate formation and growth [34]. More deposited atoms may help to grow small islands, contact with each other, and then merge into larger, but still compact islands [35,36]. Comprising nanoscale Ag crystalline grains, thicker and merged Ag nanoislands can show tight connection between the Ag crystalline grains. This compact bulk islands formation can increase the conductivity to the very high levels which need to be optimized for catalytic gas permeation processes. However, some differences can be found in case of additional annealing process. A metastable configuration tends to equilibrate through surface limited diffusion, once subjected to high temperature by annealing [37]. Because of the differences in the surface energy between the rough alumina substrate and Ag NPs, the heterogeneous interfacial formation can induce high fraction of grain boundaries, interfaces and surfaces, or residual stresses [37]. Thus, to maintain highly regular, small crystalline grains, nanosized metal island structures, a rapid thermal post-annealing process at some representative temperatures were performed with an initial film thickness of 18.50 nm. From the SEM observations in Figure 3, it is obvious that the changes in the surface morphology of Ag nanoislands accelerate with increasing annealing temperature. Figure 2a,b shows the Ag nanoparticles distribution without annealing, however a less dense surface morphology can be observed at 200 °C annealing from Figure 3a, because of the connections between Ag domains are separated after annealing process. A gradual diffusion away phenomenon was observed with increasing the temperature until 600 °C in Figure 3a–g. The Ag atoms might be diffused away from the high curvature of the grain where the surface free energy is less and it continued until an equalized surface is reached [38].

**Figure 4:**
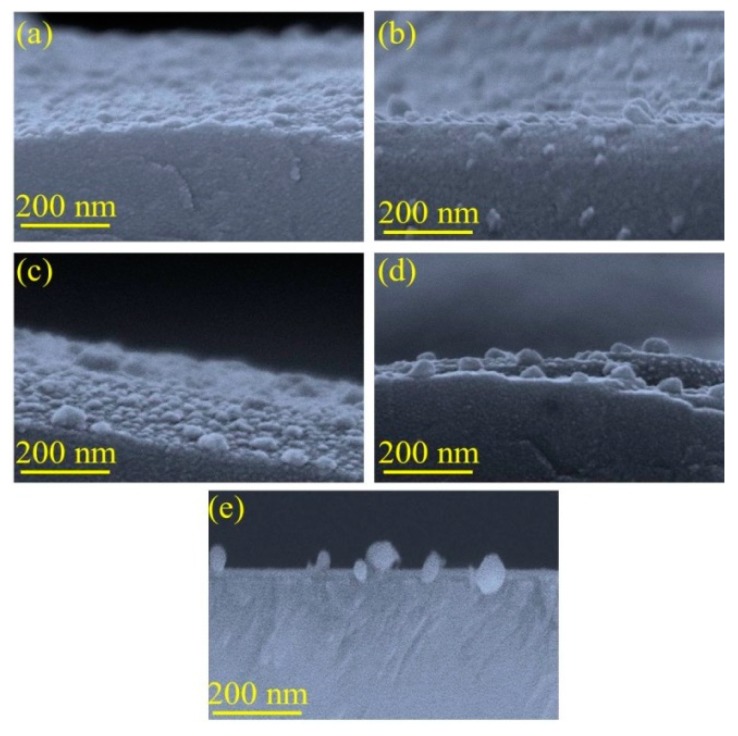
Cross sectional view of Ag nanoislands at (**a**) 200 °C; (**b**) 300 °C; (**c**) 350 °C; (**d**) 400 °C; (**e**) 500 °C.

By changing the average inter distances among the grains, more regular nanoislands formation can be seen from the respective cross section SEM observation at Figure 4a–e. When the annealing temperature is 200 °C, the nanoislands are not fully separated (Figure 4a) and a periodic island structure is visible. The surface free energy between the islands is not minimized enough to fully separate the islands [38,39]. Figure 4d shows the morphology of nanoislands at 400 °C where some nanoislands are fully separated whereas Figure 4e at 500 °C shows the formation of fully separated nanoislands. We noticed that, as the annealing temperature increased, the small particles diminish and the distances between Ag nanoislands are getting more bigger and at a temperature of 600 °C almost all Ag NPs diffuse away from the substrate (Figure 3g). This phenomenon agrees with the surface limited diffusion [34,35,36,37]. This process can be attributed to the variation of the surface diffusion coefficient with temperature [40]. Due to the differences in the chemical potentials among the Ag nanoislands, the smaller nanoislands are thermodynamically unstable and they dissolve in atoms for each fixed temperature [41]. These smaller nanoislands get incorporated by other larger nanoislands in the rough alumina substrate [42]. This temperature incorporated surface diffusion process accelerates the probability of an arriving atom finding an existing island, rather than form a new island, resulting in the decrease in the silver particle density [43]. At a temperature of 600 °C, the Ag islands volatilize from the substrate and this phenomenon will be more obvious with the increase of the temperature [44]. It is also possible that, at higher temperature the rough substrate of alumina gets acutely softer and it can’t provide a stable interface for the diffusion of Ag atoms which can be seen from Figure 3g [45]. Thus we conclude that 600 °C is high enough for the atoms of our Ag nanoislands to volatilize from the rough alumina substrate, which leads to the absence of nano-structures on the substrate.

**Figure 5:**
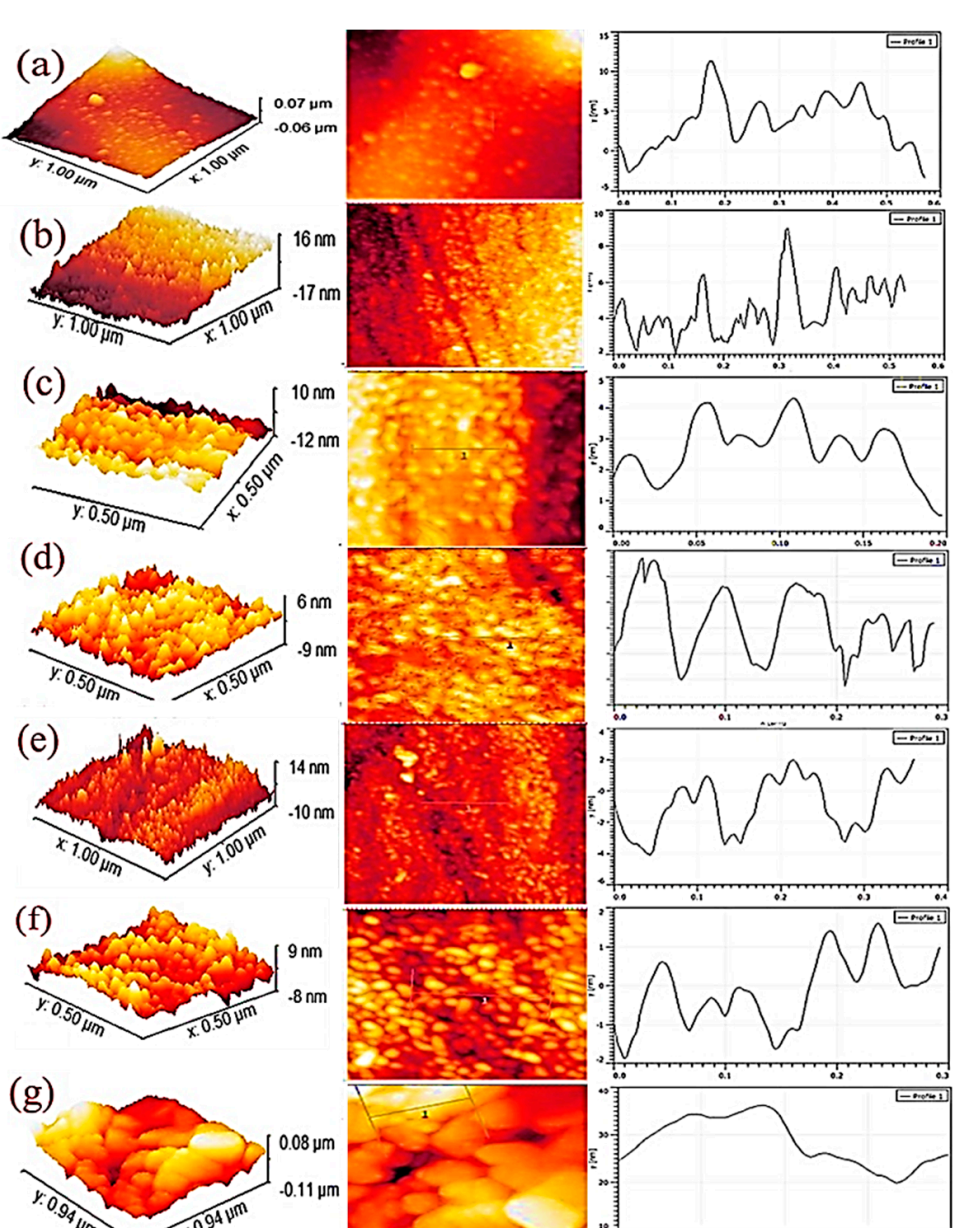
AFM analysis of Ag nanoislands (**a**) without annealing; (**b**) 200 °C; (**c**) 300 °C; (**d**) 350 °C; (**e**) 400 °C; (**f**) 500 °C; (**g**) 600 °C.

Figure 5a–g show the variation of RMS grain size of Ag nanoislands annealed at different temperatures (Table 1).

Before annealing, a RMS grain size of about 18.50 nm (Figure 5a) was measured which later showed a gradual reduction with the increasing annealing temperature. However, at 600 °C the RMS grain size shows a maximum value as all the Ag nanoislands volatize which was confirmed by SEM morphology (Figure 3g) leaving only the rough alumina substrate grains. Thus, from the AFM images, the decreasing trend of RMS grain size of Ag nanoislands (Figure 5a–f) confirms the role of the temperature dependent surface diffusion process. All the AFM measurement results are listed in Table 1.

To analyze the surface energy and hydrophobicity of the different Ag nanoisland morphologies, characterization with water droplets was carried out. The contact angle was measured according to Young’s method [46], who first proposed a minimization model of three interfacial surface energies. The contact angle images of Ag nanoislands without annealing (18.50 nm, Supplementary Figure S2a), post-annealed Ag nanoislands (Supplementary Figure S2b–e), and 200 °C annealed Ag nanoislands with Pt/Pd bimetal (Supplemenatry Figure S2f) are shown in Supplementary Figure S2. The contact angle of non-annealed Ag nanoislands shows a value of 112.5° and the contact angles of annealed Ag nanoislands keep decreasing with the increase of annealing temperature. A lowest value of 101.3° was measured for an annealing temperature of 400 °C. It is well established that, if the contact angle is higher than 90°, the surface is hydrophobic otherwise, the surface is hydrophilic. Hence, all the samples show hydrophobic properties and a maximum contact angle value of 127.3° was found after depositing 10/10 nm Pt/Pd bimetal on 200 °C thermally annealed Ag nanoislands for hydrogenation purposes (Supplementary Figure S2f). Thus, it can provide a stable performance in the H_2_ gas as water molecules can’t become stuck on the surface and decelerate the response magnitude [47]. From the AFM measurements, it was observed that, RMS grain size keeps reducing with the annealing process which also can incorporates the gradual reduction of RMS roughness (Table 1), can also be analyzed from the hydrophobicity of the samples. It has been proven that, the RMS roughness or grain size is proportional with the hydrophobicity [48]. From the water contact angle measurements, we can see the gradual reduction of the hydrophobicity with the increasing of annealing temperature (Supplementary Figure S2a–e). Additionally, the hydrophobicity is inversely proportional with the surface energy of the film surface. To determine the surface energy, the equation of Owens and Wendt and the Fowkes theory was used [48]:

$$\gamma_{SV}^{D}=(\gamma_{lV}/4)\;(\cos\theta +1{)}^{2}\;\left(\gamma_{SV}^{D}\gamma_{lV}^{D}\right){}^{1/2}+(\gamma_{SV}^{P}\gamma_{lV}^{p}){}^{1/2}=\gamma_{lV}(\cos\theta +1)/2$$

where $\gamma_{SV}^{D}$, $\gamma_{lV}^{D}$, $\gamma_{SV}^{P}$, $\gamma_{lV}^{p}$, are the dispersive and polar components of solid-vapor $\left(\gamma_{SV}\right)$ energy and liquid-vapor ($\gamma_{lV}$) energy, respectively. The surface energy calculated using this method for all the samples follows a similar fashion. The surface energy of each samples calculated by the above method are listed in Table 2. From Table 2, it can be seen that a lowest amount of surface energy (24.210 mN/m) was found for a maximum contact angle of 127.3° which agrees with the hydrophobicity and contact angle relation.

The surface energies of the samples are gradually increasing with the increasing annealing temperature as the contact angle slowly reduces. This confirms the surface diffusion process, as the surface energy keeps increasing, larger nanoislands are formed and keep separating from each other. It also confirms the AFM measurements as the RMS grain size shows a reduction trend with increasing annealing temperature.

**Figure 6:**
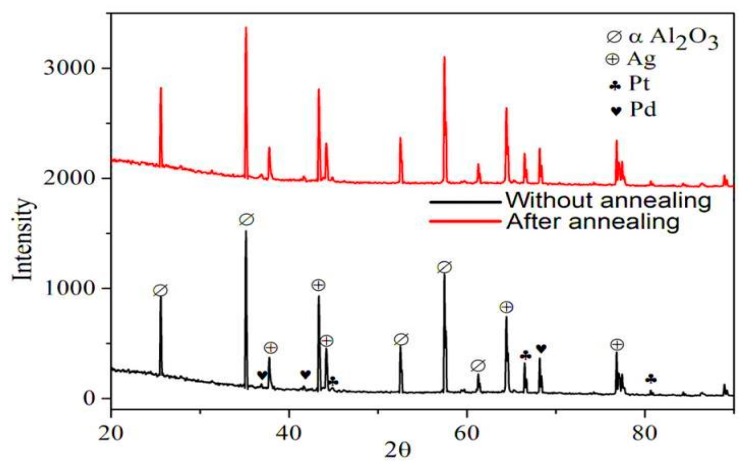
XRD analysis.

Thus, to utilize the minimum distance between the Ag nanoislands at 200 °C, we deposited the nanosized hydrogen sensing Pt/Pd bimetal which provides a percolated pathway by the hydrogen- induced lattice expansion and Coulomb scattering upon hydrogenation [36]. To confirm the Pt, Pd crystal size and their bi metal formation on Ag nanoislands (annealed at 200 °C), we conducted a high resolution XRD analysis. Figure 6 shows the XRD pattern of the Pt/Pd@Ag nanoislands for the pre- and post-annealing cases. The most intense diffraction peak at 2θ = 34.94° can be indexed to the (104) plane of α-Al_2_O_3_ [49]. In addition, the diffraction peaks observed at 2θ values of 38.26°, 44.44°, 64.66° are corresponding to the monometallic Ag (111), (200), (220) planes of the fcc crystalline structure and the peak at 2θ = 68.23° corresponds to the monometallic Pd (220) planes of the fcc crystalline structure [50,51,52,53]. A small peak originating at 2θ = 42.5° can be indexed to Pd/Ag interfacial alloy formation as it remains between the monometallic Ag peaks of 2θ = 38.26° and 44.44° while the monometallic Pd peak of 2θ = 40.15° (111) might be shifted towards higher 2θ values because of lattice mismatches formed by the Ag/Pd interface [53,54]. The intermetallic Ag/Pd peak at 2θ = 42.5° broadened by a smaller amount for the annealed Ag nanoislands at 200 °C concludes that the size reduction of Ag NPs. Peaks at 2θ = 46.3°, 67.56° are corresponding to the (200) and (220) of platinum for fcc crystal structure [53] and the diffraction peaks between the 2θ = 42.5° to 46.3° may be originated from the complex interfacial formation of Pt/Pd bimetal with Ag nanoislands. The crystallite size for Ag, Pd, Pt, was calculated by using Scherer’s formula and was found around 18.50, 10, 10 nm for each case, respectively:

β = Kλ/Dcosθ

where β is the crystallite size, λ is the wave energy of the X-ray source, D is the full width at half maximum and θ is the diffraction angle. The formations of Pt/Pd bimetal on nanostructured Ag nanoislands create a maximum hydrophobicity of 127.3° (Supplementary Figure S2f) with the lowest surface energy of 24.210 mN/m (Table 2). This lowest surface energy also leads to smaller grains and reduced inter-grain distances [36]. Additionally, upon the deposition of the Pt/Pd bimetal@Ag nanoislands on alumina, Pt capped the Pd and Ag nanoislands to form discrete bimetallic nanoparticles with high uniformity and nearly equal distances between grains. The uniform discrete manner may be formed by the lower surface energy and low polar components of the surface energy. It is well understood from the surface energy calculation (Table 2) that reduced polar components are generally more hydrophobic. Therefore, the sputtered atoms of metal attached with the surface with higher contact angle due to higher hydrophobic nature and maintain the uniformity towards the substrate [55,56]. More importantly, due to this more hydrophobic nature, sputtering atoms of the metal attached with the surface with higher contact angle and maintain the uniformity towards the substrate.

**Figure 7:**
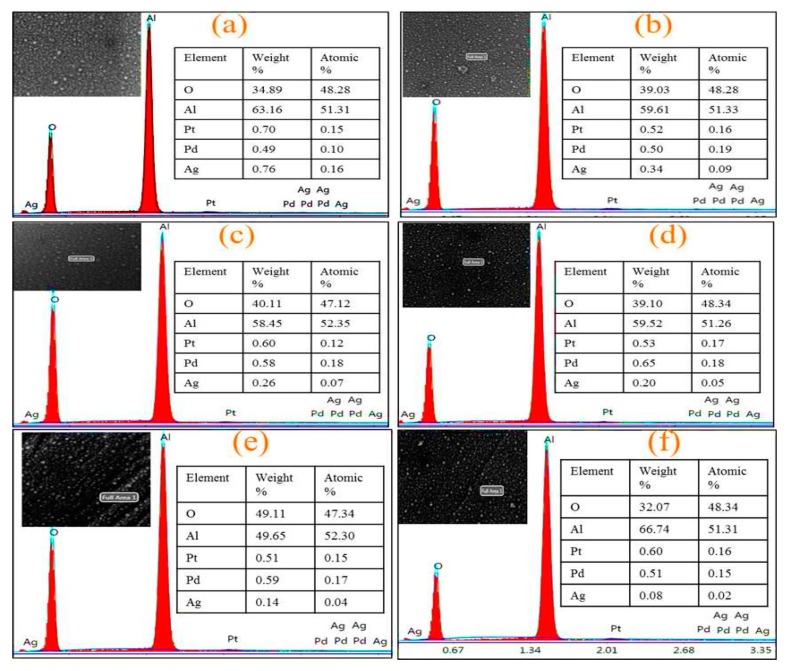
EDS elemental analysis of Pt/Pd@Ag nanoislands over alumina (**a**) without annealing; (**b**) 200 °C; (**c**) 300 °C; (**d**) 350 °C; (**e**) 400 °C; (**f**) 500 °C.

**Figure 8:**
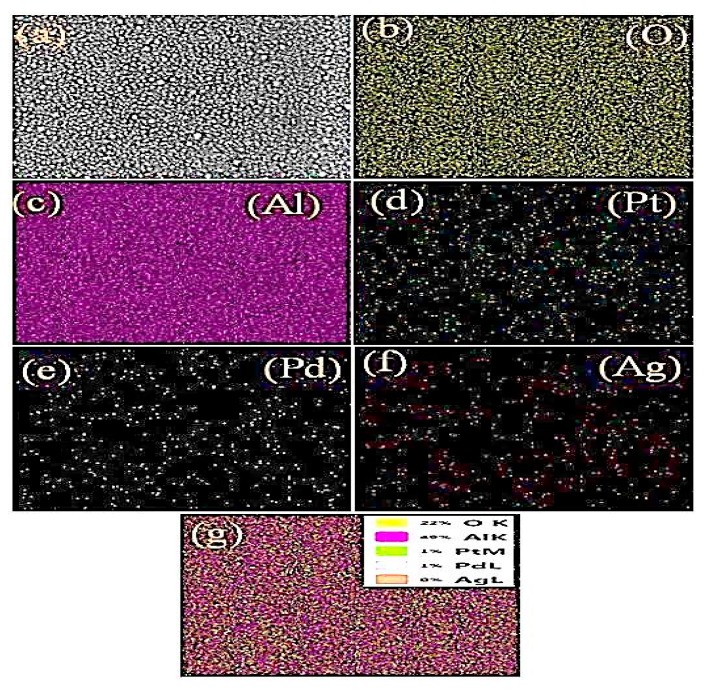
EDS mapping (surface) of Pt/Pd@Ag nanoislands over alumina substrate (**a**–**g**): samples mapping area, oxygen, aluminium, platinum, palladium, silver weight %).

**Figure 9:**
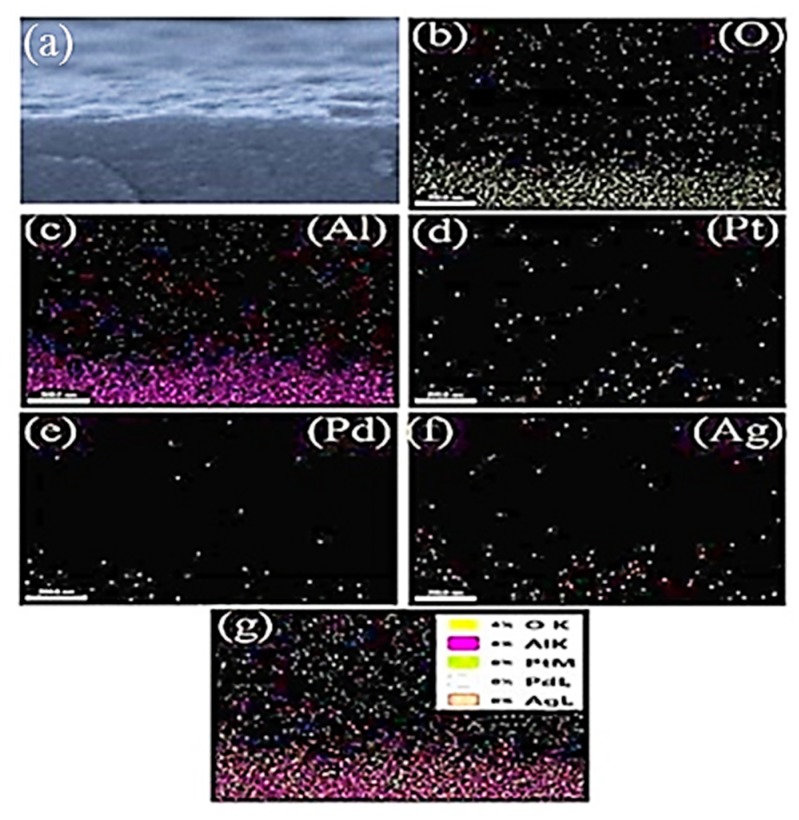
EDS mapping (cross-sectional) of Pt/Pd @Ag (200 °C annealed) nanoislands over alumina substrate (**a**–**g**): samples mapping area, oxygen, aluminium, platinum, palladium, silver weight %).

The EDS elemental composition of the as deposited Pt/Pd bimetal at different annealed Ag nanoislands on alumina (Figure 7) and an elemental mapping (surface and cross-section in Figure 8 and Figure 9) was shown. The presence of aluminum (Al), oxygen (O), silver (Ag), palladium (Pd), platinum (Pt) and the absence of peaks related to contamination concludes the formation of high-purity surface for hydrogen gas sensing. A reducing trend of Ag atomic and weight % was observed for different annealed condition as the Ag nanostructure morphology changes (Figure 7). A surface and cross-sectional elemental mapping for the sample (Pt/Pd@Ag nanoisland on alumina at 200 °C) was shown in Figure 8 and Figure 9 for observing the uniform nanoparticle distribution over the entire substrate. From the surface and cross-sectional EDS mapping, it can be observed that Pt/Pd and Ag are distributed homogeneously over the substrate, which further confirms the formation of a highly uniform Pt/Pd bimetallic thin film on the Ag nanoislands.

**Figure 10:**
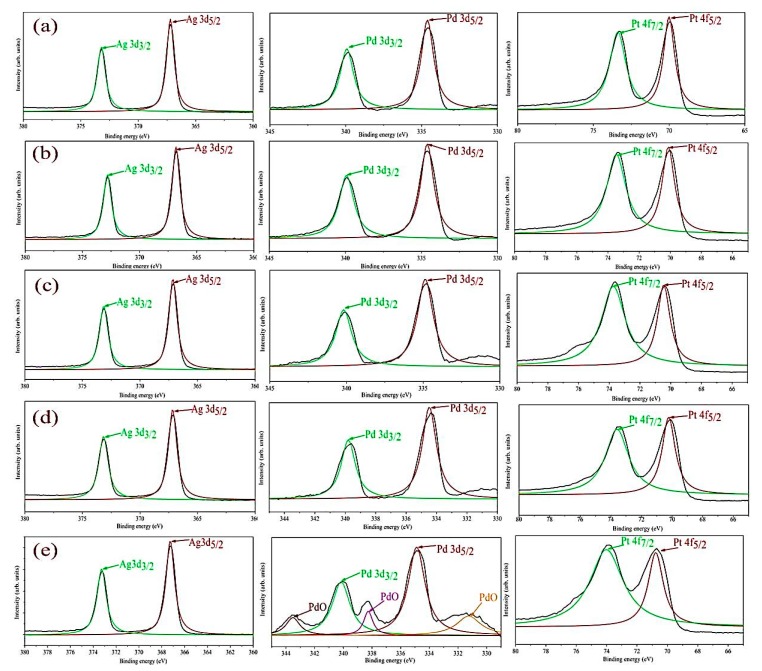
XPS analysis of Pt/Pd @ Ag nanoislands Surface morphology at (**a**) 200 °C; (**b**) 300 °C; (**c**) 350 °C; (**d**) 400 °C; (**e**) 500 °C.

Figure 10 shows the XPS analysis for examining the surface compositions of the Pt/Pd bimetal with the Ag nanoislands at different annealing conditions. The morphological differences among the nanostructured Ag islands for surface diffusion coefficient at different temperature causes the interfacial composition to change for metal alloys. The corresponding doublets of Ag 3d, Pd 3d, Pt 4f core levels were de-convoluted into Ag 3d_5/2_, Ag 3d_3/2_, Pd 3d_3/2_, Pd 3d_5/2_, Pt 4f_5/2_, Pt 4f_7/2_ by the Gaussian-Lorentzian fitting method after linear background subtraction. A maximum shift in higher binding energies for Ag 3d_5/2_ = 368 eV, Ag 3d_3/2_ = 374 eV Pd 3d_3/2_ = 340.5 eV, Pd 3d_5/2_ = 335, Pt 4f_5/2_ = 70.5, Pt 4f_7/2_ = 74 for the sample Pt/Pd@Ag nanoislands annealed at 200 °C was observed (Figure 10b). These slight shifts can be promoted from the electronic interaction and maximum interfacial alloy formation between the Pt/Pd bimetal and Ag nanoislands due to the differences in electron negativity [56,57]. The XPS measurements (Table 3) confirm that the bimetal alloys are tightly connected with the Ag nanoislands as the temperature diffusion induced separations are smaller for the 200 °C and 300 °C cases which induce additional lattice strain or a higher level of electron donation from the bimetal [57,58]. All the measurements are summarized in Table 3. Thus, a percolated pathway is constructed within the Ag nanoislands and remains strongly connected which can improve the surface conductivity.

**Figure 11:**
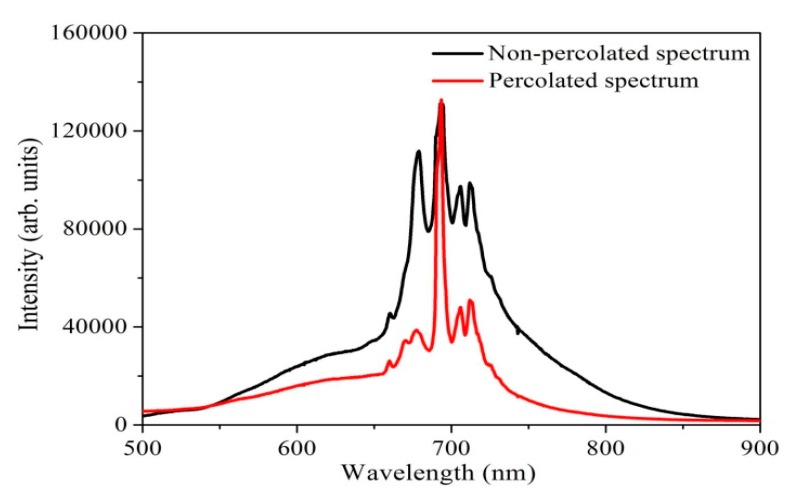
Photoluminescence spectroscopy.

Figure 11 illustrates the photoluminescence of Pt/Pd bimetal distribution on the annealed (200°) and non-annealed Ag nanoislands. A higher intensity of the photoluminescence was observed from the non-annealed Pt/Pd@Ag nanoislands as the larger amount of Ag (18.50 nm) is covered with oxygen which increases the defect states [59,60,61]. It can be possible that, because of the higher amount of silver atoms (Figure 7a) and larger nanoislands; Pt/Pd bimetal can’t fully cover the nanoisland surface. Thus, a non-percolated surface is formed which shows higher oxidized defect states in photoluminescence. Whereas, the post annealed sample case, the intermixing alloy formation among the metals is good (Figure 10b) show a less intense photoluminescence due to the uniform capping of Pt/Pd bimetal. A quantum confinement effect (QCE) is a possible mechanism where the Ag nanoislands (6.50 nm) with Pt/Pd bimetal show less oxidized defect states that further confirms the tightly connection and good capping of Pt/Pd bimetal over the Ag nanoislands [21,62,63,64]. In this case, a highly percolated pathway can be formed that can increase the synergetic effects of the metal alloys [65].

### 3.2. Hydrogenation and Dehydrogenation Studies

In the synthetic air environment, Hydrogen molecules physisorb on the Pd surface and gradually dissociates into hydrogen atoms chemisorbs in the Pd lattice structure for forming the Pd-hydride (Pd-H) as follows:

H_2_ (g) → 2H (ads)

Pd + H (ads) → Pd-H

**Figure 12:**
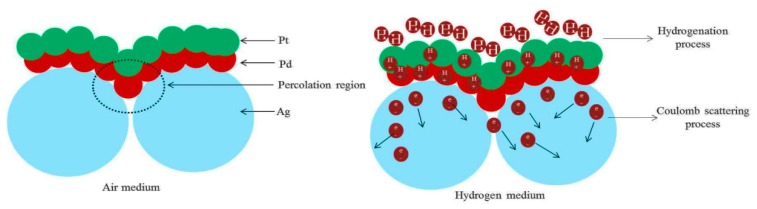
Percolated pathway formation and hydrogenation mechanism (from left to right).

Thus the dissociated hydrogen atoms transform the metal to a metal hydride phase by expanding the volume of the Pd lattice structure which narrows the gaps between the neighboring clusters of nanoparticles [29,30]. Along with the reduced gaps, the base resistance between nanoislands decreased. However, the resistivity response of Pd-H is almost a factor of 2 higher than that of pure Pd metal [66]. At the time of hydrogen molecules chemisorption process, a Coulomb scattering process increases the positive charge accumulation, thus enhances the metal hydrides resistivity [29,30,66,67]. In an air environment, the presence of oxygen enables the formation of catalytic water molecules at the Pd surface that impedes surface coverage for chemisorbed hydrogen available to be absorbed [68,69,70]. This phenomenon decreases the Pd adsorption sites, lowering the hydrogen physical absorption [71]. Thus, it also extends the time required for Pd lattice structure to host hydrogen molecules at α-phase and transforming to Pd-H into β-phase which eventually slows down the whole hydrogenation process [72]. Alloying the Pd lattice with Pt could change the surface chemistry as a favorable means in the aforementioned situation [73]. It was also reported that Pt is a better catalyst for hydride formation which is the rate limiting reaction for hydrogenation response for an optimum thermal condition (≥100 °C) [74,75]. Thus, optimizing the temperature and alloying with Pt can be an effective way to accelerate the hydrogenation performance of Pd thin film [72,73,74,75]. However, along with optimizing temperature, the catalytic high surface area-based Ag nanoislands can enhance Pt/Pd bimetal’s synergetic effect. Highly regular Ag nanoisland formation (Figure 4b) on the rough alumina substrate increases the surface to volume ratio which accelerates the interfacial alloy formation with the catalytic bimetal (Figure 10b). In addition, the complex alloy formation may increase the response of hydrogenation permeation by increasing the active surface coverage. Furthermore, it was found that Ag alloying with Pd can be advantageous for the embrittlement and mechanical issue of Pd lattice which might be more improved with the Pt/Pd@Ag nanoislands case [76,77]. A higher surface to volume ratio-based Ag nanoislands enhances the bimetal capping configuration that makes a percolation pathway that is suitable for higher carrier mobility. It is believed that a hydrogen induced lattice expansion in nanosized catalytic sputtered bimetal may form a shorter conduction pathway for carrier mobility in the transduction process between physisorption to chemisorption on an insular substrate [78,79]. However, maintaining the homogenous nanosized bimetal capping structures is highly challenging, and a catalytic metal nanoislands structure beneath the bimetal structure increases the connection between the metal atoms and enhances the surface conductivity which may enhance the hydrogen induced lattice expansion faster. The induced hydrogen may scatter all the carrier charges by Coulomb scattering process which involves a charge variation in the catalytic metal alloys [71,73]. The schematic of the formation of percolated bimetal based on Ag nanoislands (Pt/Pd@Ag NI) and its hydrogenation process are shown in Figure 12.

**Figure 13:**
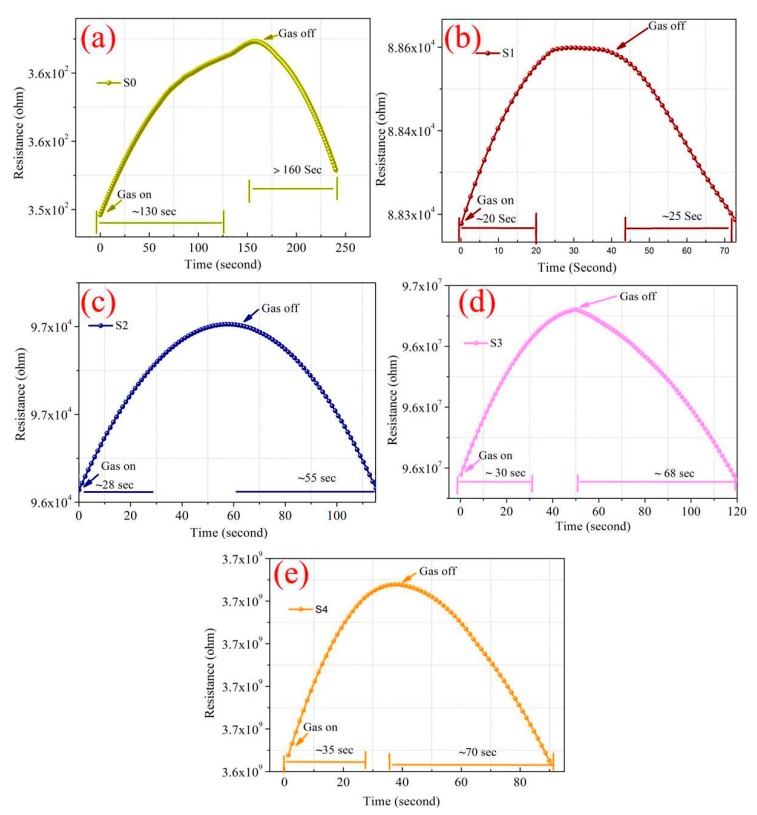
Response and recovery time of (**a**) Pt/Pd @ non-annealed Ag nanoislands; (**b**) Pt/Pd @ 200 °C annealed Ag nanoislands; (**c**) Pt/Pd @ 300 °C annealed Ag nanoislands; (**d**) Pt/Pd @ 350 °C annealed Ag nanoislands; (**e**) Pt/Pd @ 400 °C annealed Ag nanoislands.

In Figure 13a–e, we observe a close relation between the base resistance and Ag nanoislands diffusion process, where a range of kΩ to GΩ was measured depending on the separated distance between the islands (Figure 4) with Pt/Pd (10/10 nm) bimetal capping layer. An optimized conductivity is necessary for the catalytic alloy formation and the surface energy [72]. Additional controlled experiments such as, thinner Pd (10 nm) on alumina, thinner Pt (10 nm) on alumina, thinner Pt@Pd on (10/10 nm) on alumina, thicker Pt@Pd on (40/40 nm) on alumina, thinner Ag@Pt (18 nm Ag annealed at 200 °C with 10 nm Pt) on alumina, thinner Ag@Pt (18 nm Ag annealed at 200 °C with 10 nm Pd) on alumina were analyzed (Supplementary Figure S1a–f) to ensure the enhanced synergetic effects of the percolated Pt@Pd bimetal on nanostructured Ag islands. A thinner (~10 nm) Pd thin film deposited by RF magnetron sputtering on alumina, showed a response and recovery time of 60/250 s at 120 °C (1% H_2_) with a surface resistivity of average 82 GΩ (Supplementary Figure S1a). The high amount of resistance might be due to the loose connection of Pd nanoparticles on the alumina substrate. We believe that smaller sputter deposited Pd nanoparticles will increase the surface resistivity and slower the response and recovery time. Hence, a slower response and recovery time was observed because of non-percolated nanoparticles that may not strongly contribute to the hydrogen induce lattice expansion [12]. Higher size of (>10 nm) of Pd nanomaterials were not checked as it may considerably increase the response and recovery property [29,65]. Similar kinds of properties in thinner Pt nanoparticles (~10 nm) deposited alumina was observed, where an average surface resistivity of 130 MΩ and a slower response time of 150 s with not full recovery towards 1% hydrogen gas (Supplementary Figure S1b) was recorded. Pt@Pd bimetal with different sizes such as thinner (10/10 nm) and thicker (40/40 nm) were exposed in 1% hydrogen (Supplementary Figure S1c,d) gas to analyze their hydrogenation and dehydrogenation properties at 120 °C. A considerably shorter response and recovery time (43/55 s) in case of 10/10 nm size Pt@Pd in comparison to single layered thinner Pd or Pt was observed at 1% hydrogen gas (120 °C) which is due to the enhanced synergetic effects of the bimetal formation [31]. We also carefully fabricated different Ag@Pd, Ag@Pt (18/10 nm) thinner bilayer structures for analyzing the synergetic effect towards 1% hydrogen gas (120 °C) where Ag@Pd (thinner) showed better catalytic response than Ag@Pt as the diffusion of hydrogen atoms in Pd is more higher (Supplementary Figure S1e,f) [28,31]. However, their response and recovery was quite slow in comparison to a 200 °C annealed Ag nanoislands-Pt/Pd (S1) sample (Figure 13b) [49,72]. Meanwhile, bare Ag nanoparticles don’t show any catalytic response towards hydrogen gas [32]. However, Ag nanoparticles alloyed with highly catalytic Pd nanoparticles increase the synergetic properties toward hydrogen absorption and desorption which is shown by Sharma et al. [32,77].

**Figure 14:**
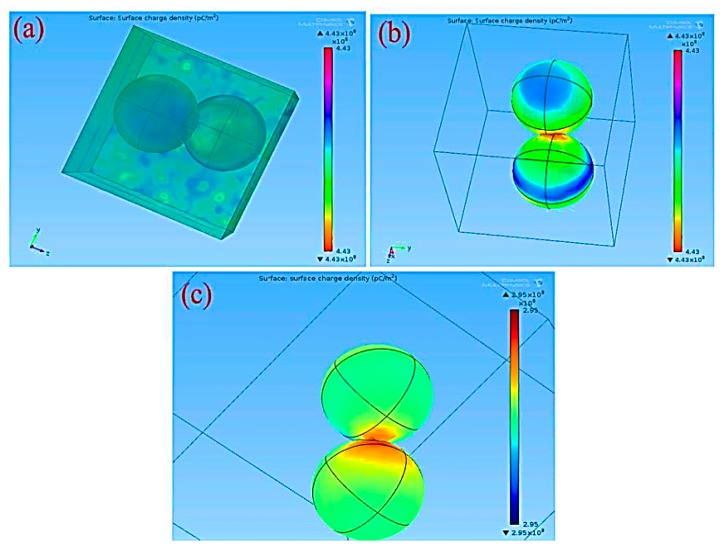
Computational analysis of Pd/Pt (5/5 nm) bimetal (**a**) Surface charge density at the interface of bimetal; (**b**) at air environment; (**c**) at hydrogen environment.

**Figure 15:**
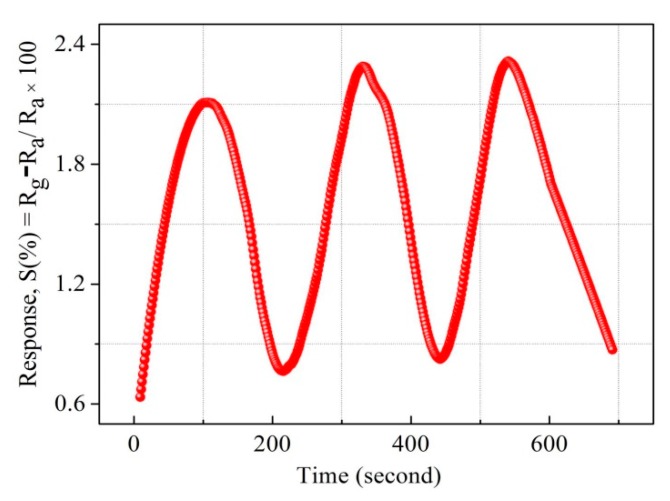
Repeatability of S1 at 10,000 PPM (120 °C).

We also analyzed the Pt/Pd bimetal thickness effect and their charge density. A computational analysis was done using COMSOL Multiphysics simulator to optimize the Pt/Pd size and their charge density in the interfacial alloy (Figure 14). The simulation results are summarized in Supplementary Figure S3. The simulation was performed up to 15 nm for Pt and 10 nm for Pd, as a thicker Pt layer can form a barricade to the injection of the hydrogen molecules to the Pd surface; which may impede the sufficient hydride formation for Pd; furthermore, the hydrogen-induced expansion of Pd might be hindered due to the excessive strain imposed by the Pt capping layer, and the catalytic water formation at the Pt surface can be enhanced, hence slowing down the hydrogenation response [56,69]. Figure 14a–c illustrate the distribution of electrical permittivity of the Pt/Pd bimetal (10/10 nm) and reveal that a certain applied potential difference on the boundaries of the chamber creates a surface charge density depending on the medium (air, hydrogen) in the chamber inside. A highly dense surface charge density (Figure 14a,b,c) can be observed from the interface of Pt/Pd bimetal as the relative permittivity of Pt/Pd bimetal is much higher ($\varepsilon_{r}=67.88$) in comparison to Pt ($\varepsilon_{r}=17.19$) and Pd ($\varepsilon_{r}=13.69$) [79]. We found a maximum amount of surface charge density for Pt/Pd (10/10 nm) case in air environment and a lowest value in hydrogen environment from all other thickness variation (Supplementary Figure S3). This result concludes that, a maximum amount of hydrogen physisorption and chemisorption might occur in case of uniform 10/10 nm Pt/Pd bimetal case, hence effectively increases the electrical resistance of the bimetal. To relate this simulated analysis with practical applications, we fabricated 10/10 nm Pt/Pd@Ag nanoislands (annealed at 200 °C) on alumina substrate and Pt/Pd@Ag annealed at 200 °C sample shows faster response and recovery of 20/25 s at 1% hydrogen gas (Figure 13b) and a maximum amount of 2.3% response magnitude (120 °C) was observed (Figure 15). Whereas very slow response and recovery was observed for other samples (Figure 13c–e) which could happen for several factors such as the Ag nanoisland diffusion process, Pt/Pd/Ag alloy formation, gaps in nanoparticles, etc.

**Figure 16:**
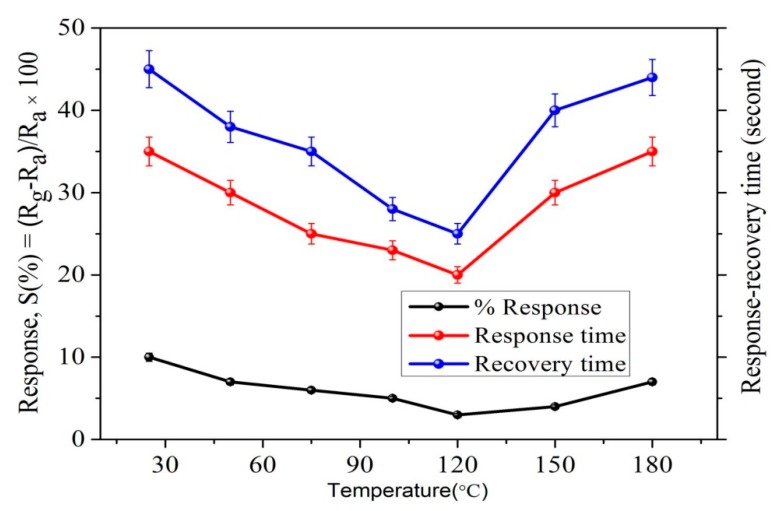
Response and recovery at different temperature (10,000 ppm hydrogen).

**Figure 17:**
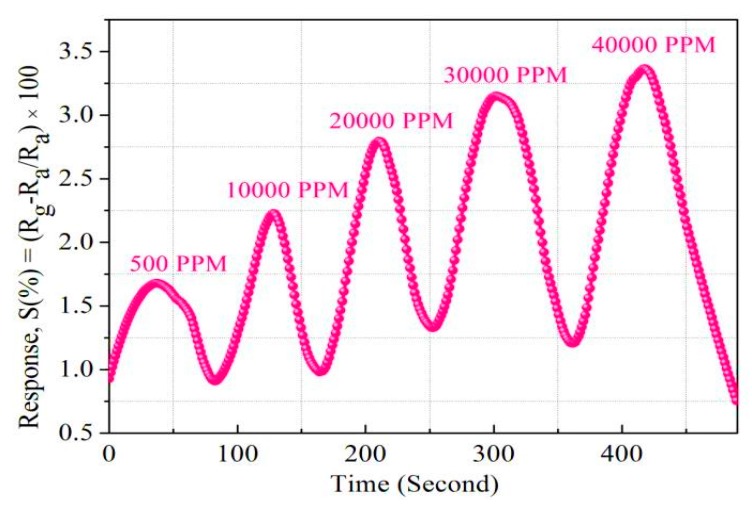
Response magnitude at different hydrogen concentration for S1 sample at 120 °C.

From Figure 3 and Figure 4 (SEM surface and cross-section), the inter-distances between the Ag nanoislands can be observed while the formation of Pt/Pd bimetal capping layer on the smallest inter-distance based (Ag annealed at 200 °C) Ag nanoislands show a higher inter-molecular interaction which was observed in XPS analysis (Figure 10a). The increased amount of eV in Ag 3d_3/2_, Ag 3d_5/2_, Pd 3d_3/2_, Pd 3d_5/2_, Pt 4f_5/2_, Pt 4f_7/2_ (Table 3) agrees with the strong inter-molecular interaction in case of the 200 °C annealed Ag nanoisland Pt/Pd (10/10 nm) samples case, whereas the reduction in the eV for other samples conclude lesser amount of interfacial morphology that agrees with the hydrogen response and recovery properties in Figure 13c–f. The lesser amount of interfacial morphology can be understood from Figure 5 and Figure 7 (AFM, EDS elemental analysis) where a reduction in the RMS grain size of the nanoislands (Table 1) and weight % was observed. These observations agree with the SEM surface and cross-sectional analysis (Figure 3 and Figure 4). We believe the smallest inter-distance-based Ag nanoislands/Pt/Pd with good interfacial catalytic structure enhances the synergetic effects towards hydrogen gas absorption and desorption. Due to this strong interfacial surface morphology, highly optimized percolated channels form which may eventually increase the Coulomb scattering process (Figure 13). Thus, a faster hydrogen absorption and desorption occurred in the S1 sample. Figure 13 shows the hydrogenation and dehydrogenation curves (resistance versus time) for Pt/Pd@Ag nanoislands at 10,000 ppm hydrogen at 120 °C temperature for the different nanoisland structures. Gradual changes in the base resistance were observed for the different annealed condition of Ag nanoislands (Figure 13a–e) depending on the nanoisland morphological structure. The time needed for 90% change in the base resistance after exposure to hydrogen gas and the time needed for the resistance to reach its initial value are defined as response and recovery time. A longer average time of 130 s was measured as response time for the Pt/Pd@Ag nanoislands non-annealed sample (labeled as S0) and a sluggish recovery of more than average 160 s at 120 °C. Lesser amount of catalytic metal alloy interface could be a probable reason for this. However, a faster response time of average 20 s and recovery time of average 25 s at 120 °C for Pt/Pd@Ag nanoislands annealed at 200 °C sample (labeled as S1), which might be a good and better catalytic metal alloys interfacial effect along with uniform particle distribution. Apart from S1 sample, S2, S3 and S4 show little delayed response and recovery time (Figure 13) along with higher base resistances depending on the Ag nanoislands morphology. The observations conclude that connectivity between bimetal nanoparticles and metal islands is reducing with the diffusion process induced by annealing temperature. Hence, a less catalytic metal alloy interface and synergetic interplay are occurring which delaying the hydrogen physisorption and chemisorption process. Eventually, for the S1 sample case, the interactions of hydrogen molecules with the interstitial sites of catalytic metals alloy scatters the electrons faster to incorporate hydrogen induced lattice expansion at the β (metal hydride phase) which eventually shows a faster response time [67]. Along with faster response, a faster recovery time for S1 sample could be due to the higher grains of the interface that can be used as desorption pathway for hydrogen molecules [67]. In addition with the interfacial grains, an optimized temperature of 120 °C was the best fit of electron scattering process as, Pt has higher absorption rate at 100 °C with fast response but lacks in recovery whereas, Pd has slower absorption rate along with high response magnitude and faster recovery [73,79,80]. Furthermore, catalytic metal alloy of Ag needs a moderate elevated temperature to dissociate the chemisorbed hydrogen molecules [65,66]. A co-operative interaction between the bimetal and metal islands layer induce more tensile stress that helps to dissociate the molecules faster [73,74,75]. So it can be assumed that, at 120 °C both the bimetal and nanoislands metal compete each other and incorporate a faster response and recovery time. Along with good catalytic metal interface, a highly hydrophobic surface is desired because the presence of oxygen enables catalytic water formation at the Pd surface, thereby reducing the steady-state surface coverage of the chemisorbed hydrogen available to be observed [72]. Sample S1 shows a water contact angle of 127.3° (Supplementary Figure S2f) which confirms the hydrophobicity of the sensing surface. Furthermore, the selectivity of the S1 samples for different gases showed in Supplementary Figure S4. Figure 16 summarizes the response, recovery time and response magnitude for 1% hydrogen gas exposure at different temperature. The response magnitudes of S1 sample with various hydrogen concentrations (500 ppm to 40,000 ppm) at 120° are illustrated in Figure 17.

**Figure 18:**
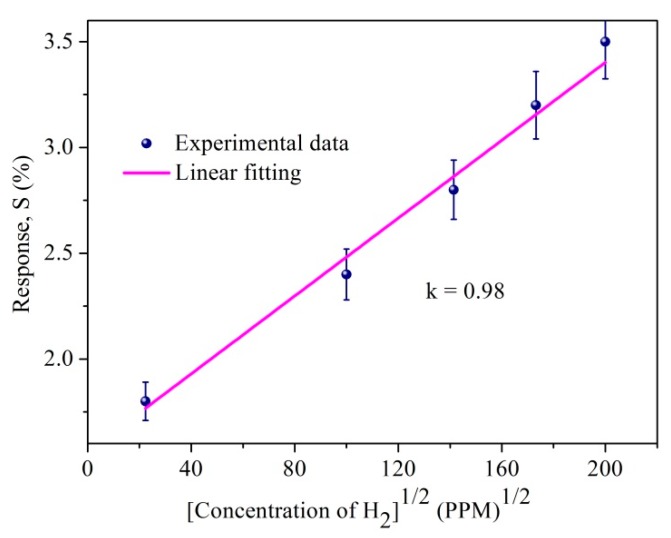
Sievert’s law for S1 sample at 120 °C.

The response magnitude for wide ranges of hydrogen concentration was theoretically modeled by Sievert’s law for S1 sample in Figure 18, where linearity between the response and the square root of hydrogen concentrations was observed. The linearity confirms that sample S1 follows Sievert’s law S (%) α √P(H_2_), where P(H_2_) is the partial pressure of hydrogen [67]. Hydrogen absorption in Pd materials, such as thin films, nanowires, and nanodisks [74,75,76,77,78,79,80], has shown hysteresis and breaking phenomena after a few cycles of hydrogen absorption whereas, the presently studied Pt/Pd@Ag nanoisland structure performed well at the high concentration of 40,000 ppm hydrogen. A negligible hysteresis effect (Figure 15) was due the uniform Pt layer which enhances the passing of hydrogen molecules to interact with Pd/Ag alloy along with an increased clamping effect that reveals a tensile stiffness of a laminate specimen is in inverse relation to the thickness of the specimen [79,80]. Figure 15 illustrates the repeatability of the as fabricated S1 device with a constant response magnitude of 2.3% at a concentration of 10,000 ppm hydrogen at 120 °C.

The as-prepared device (S1) has the potential of the sensing of hydrogen gas in real field applications although it can be strongly affected by the presence of water vapor [67,73]. This can slow down the hydrogenation response by impeding the dissociation of hydrogen molecules on the catalytic metal surface [69,70,71]. However, in this work a highly hydrophobic surface having a contact angle of 127.3° (Supplementary Figure S2f) can create more active sides for hydrogen adsorption by impeding the water vapor. Thus, it creates a greater hydrogenation and dehydrogenation property by increasing the hydrophobicity for quantum size Pt/Pd@Ag nanoislands structure.

## 4. Conclusions

In this current study, the hydrogenation and dehydrogenation properties of Pt/Pd bimetal decorated over Ag nanoislands on an alumina substrate were studied. Thermally annealing process-induced surface diffusion provides a high surface to volume ratio-based Ag nanostructured island morphology which provides an enhanced catalytic property with Pt/Pd bimetal for the adsorption and desorption of hydrogen molecules at an elevated (120 °C) temperature. A response magnitude of 2.3% at 10,000 ppm hydrogen gas exposure and a detection range of 500 to 40,000 ppm for hydrogen gas were observed with a good repeatability at 10,000 ppm H_2_. A fast response and recovery time of 20/25 s for 10,000 ppm at 120 °C were recorded for the material, which also shows high hydrophobicity. We expect that the as prepared (Pt/Pd@Ag nanoislands) device will be a promising building block for future hydrogen detection technology in hydrogen economy-based applications.

## Figures and Tables

**Table 1:** AFM measurements.

Sample	RMS Grain Size (nm)
Ag@alumina (non-annealed)	18.50
Ag@alumina at 200 °C	6.5
Ag@alumina at 300 °C	4.96
Ag@alumina at 350 °C	2.63
Ag@alumina at 400 °C	2.45
Ag@alumina at 500 °C	1.76
Ag@alumina at 600 °C	30

**Table 2:** Contact angle and surface energy measurements.

Sample	Contact Angle	$\gamma_{SV}\;(\mathrm{mN}/m)$	$\gamma_{SV}^{D}\;(\mathrm{mN}/m)$	$\gamma_{SV}^{P}\;(\mathrm{mN}/m)$
Ag@alumina (non-annealed)	112.5°	28.79	28.11	0.68
Ag@alumina at 200 °C	106.7°	28.72	28.575	0.14
Ag@alumina at 300 °C	105°	29.88	29.72	0.16
Ag@alumina at 350 °C	104.2°	31.78	31.44	0.34
Ag@alumina at 400 °C	101.3°	32.816	32.58	0.236
Pt/Pd@200 °C annealed Ag nanoislands on alumina	127.3°	24.210	22.128	2.08

**Table 3:** XPS measurements.

Sample	Ag 3d_3/2_ (eV)	Ag 3d_5/2_ (eV)	Pd 3d_3/2_ (eV)	Pd 3d_5/2_ (eV)	Pt 4f_5/2_ (eV)	Pt 4f_7/2_ (eV)
Pt/Pd@ non-annealed Ag nanoislands on alumina	373.5	367.5	340	334.5	70	73.5
Pt/Pd@200 °C annealed Ag nanoislands on alumina	374	368	340.5	335	70.5	74
Pt/Pd@300 °C annealed Ag nanoislands on alumina	373.8	367.7	340.5	334.8	70.2	73.7
Pt/Pd@350 °C annealed Ag nanoislands on alumina	373.5	367.3	340	335	70	73.8
Pt/Pd@400 °C annealed Ag nanoislands on alumina	373	367.4	340	335	70	74

## Supplementary Materials

The following are available online at http://www.mdpi.com/1424-8220/19/1/86/s1, Figure S1. Response recovery characteristics of different catalytic materials at 10000 ppm hydrogen gas (120 °C) (a) Pd (10 nm) on alumina (b) Pt (10 nm ) on alumina (c) Pt@Pd on (40/40 nm) on alumina (d) Pt@Pd on (10/10 nm) on alumina (e) Ag@Pt (18 nm Ag annealed at 200 °C with 10 nm Pt) on alumina (f) Ag@Pt (18 nm Ag annealed at 200 °C with 10 nm Pd) on alumina; Figure S2. Water contact angle of Ag nanoislands (a) without annealing, (b) 200 °C, (c) 300 °C, (d) 350 °C, (e) 400 °C, (f) Pt/Pd @ Ag nanoislands; Figure S3. Bimetal size vs. surface charge density; Figure S4. Selectivity.
