# Supplementary material for: Fast Hydrogenation and Dehydrogenation of Pt/Pd Bimetal Decorated over Nano-Structured Ag Islands Grown on Alumina Substrates
Source: Sensors (Basel). 2018 Dec 27;19(1):86. doi: 10.3390/s19010086 (PMC6339134; doi:10.3390/s19010086)
Supplement: Supplementary file 1 [file sensors-19-00086-s001.pdf]

Supplementary Material

Fast Hydrogenation and Dehydrogenation of Pt/Pd Bimetal Decorated over Nano-Structured Ag Islands Grown on Alumina Substrates

Md Habibur Rahaman, Usman Yaqoob and Hyeon Cheol Kim *

School of Electrical Engineering, University of Ulsan, 93 Daehak-ro, Nam-gu, Ulsan 44610, Korea; habibiiuceee@gmail.com (M.H.R.); usmanyqb3@gmail.com (U.Y.)

*Correspondence: hckim08@mail.ulsan.ac.kr; Tel.: +821085551426, +82-52-259-2199

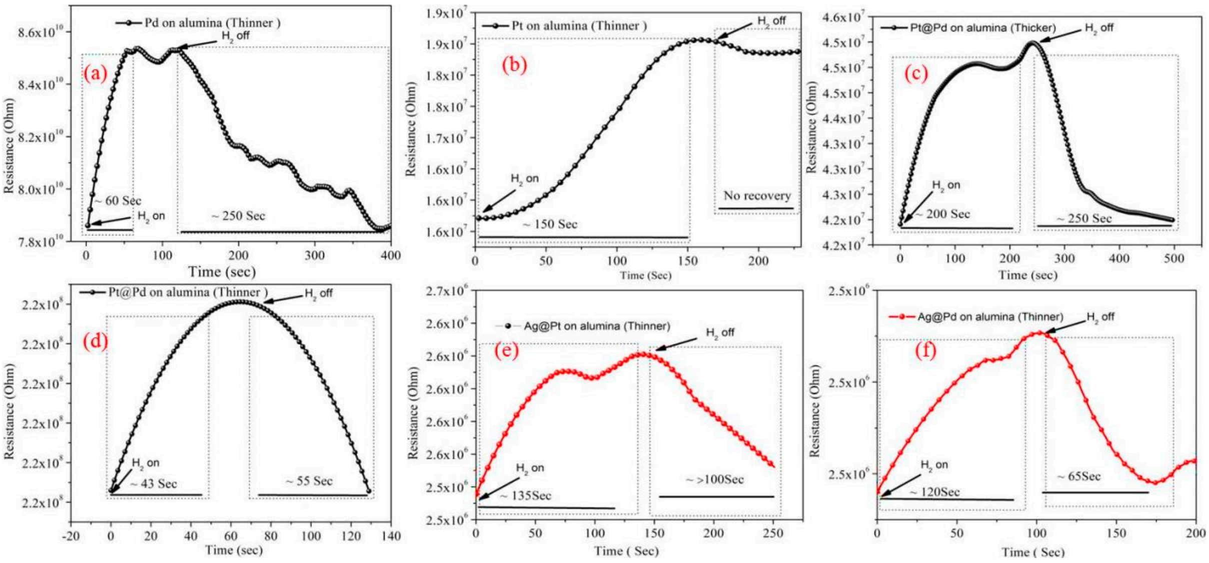

Figure S1. Response recovery characteristics of different catalytic materials at 10000 ppm hydrogen gas (120˚C) (a) Pd (10 nm) on alumina (b) Pt (10 nm) on alumina (c) Pt@Pd on (40/40 nm) on alumina (d) Pt@Pd on (10/10 nm) on alumina (e) Ag@Pt (18 nm Ag annealed at 200˚C with 10 nm Pt) on alumina (f) Ag@Pt (18 nm Ag annealed at 200˚C with 10 nm Pd) on alumina.

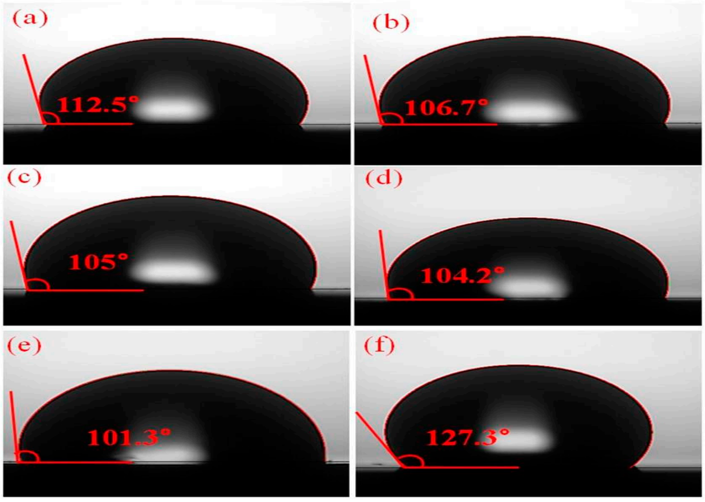

Figure S2. Water contact angle of Ag nanoislands (a) without annealing, (b) 200°C, (c) 300°C, (d) 350°C, (e) 400°C, (f) Pt/Pd @ Ag nanoislands.

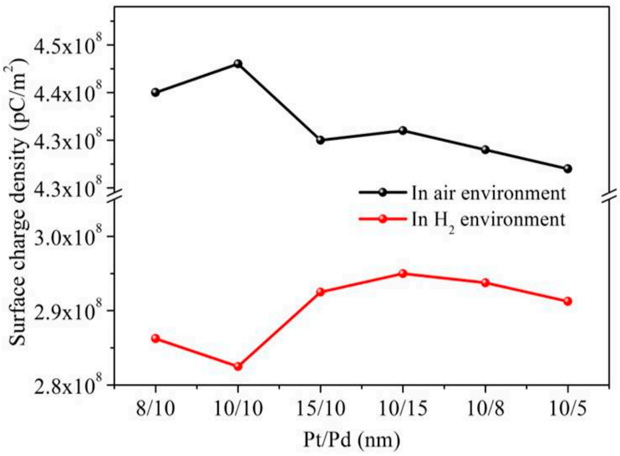

Figure S3. Bimetal size vs. surface charge density.

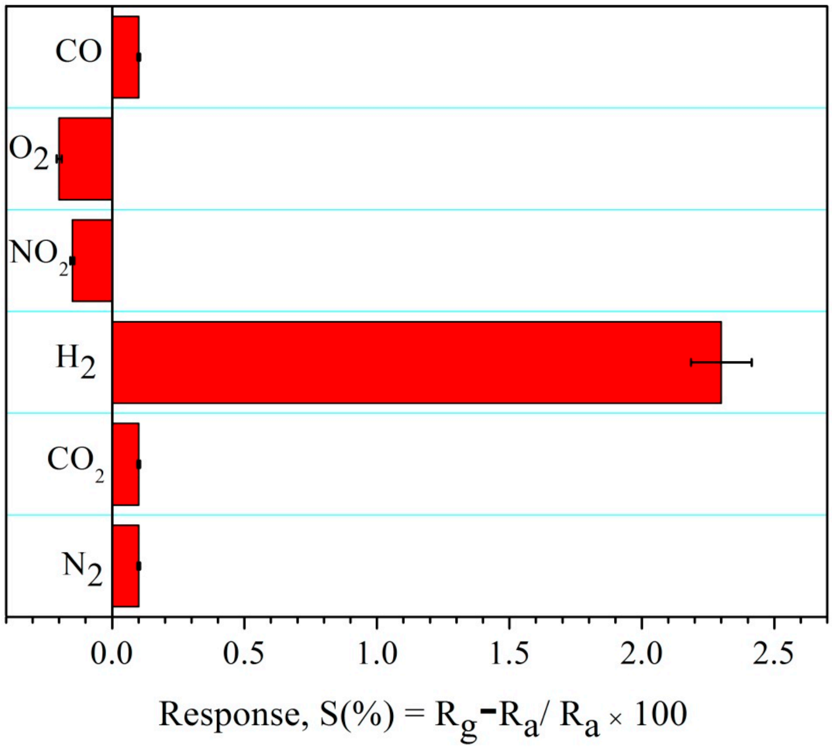

Figure S4. Selectivity
